# Effect of ambient temperature on outpatient admission for osteoarthritis and rheumatoid arthritis in a subtropical Chinese city

**DOI:** 10.1186/s12889-021-11994-0

**Published:** 2022-01-25

**Authors:** Desheng Zhao, Jian Cheng, Ping Bao, Yanwu Zhang, Fengjuan Liang, Hao Wang, Xu Wang, Shiyuan Fang, Hong Su

**Affiliations:** 1 Department of medical administration, The First Affiliated Hospital of USTC, Division of Life Sciences and Medicine, University of Science and Technology of China, Hefei, 230001 Anhui China; 2 Department of Epidemiology and Biostatistics, School of Public Health, Anhui Medical University, Hefei, 230032 Anhui China; 3 Nursing Department, The First Affiliated Hospital of USTC, Division of Life Sciences and Medicine, University of Science and Technology of China, Hefei, 230001 Anhui China; 4 Information Center, The First Affiliated Hospital of USTC, Division of Life Sciences and Medicine, University of Science and Technology of China, Hefei, 230001 Anhui China; 5 Anhui Provincial Children’s Hospital, Hefei, Anhui Province China

**Keywords:** Temperature, Osteoarthritis, Rheumatoid arthritis, Admission

## Abstract

**Background:**

Current findings on the impact of weather conditions on osteoarthritis (OA) and rheumatoid arthritis (RA) are sparse and not conclusive. This study aimed to investigate the relationship between temperature change and OA/RA admission.

**Methods:**

Daily OA/RA admission, meteorological data and pollutants from 1 January 2014 to 31 December 2017 in Hefei, China, were collected. We quantified the relationship between ambient temperature and OA/RA admission using a distributed lag nonlinear model (DLNM). Stratified analyses by gender and age were also examined.

**Results:**

Temperature decrease was significantly associated with RA admission (25th percentile of temperature versus 50th percentile of temperature), with the acute and largest effect at current days lag (RR: 1.057, 95%CI: 1.005–1.111). However, no significant association between temperature and OA admission was observed. When conducting subgroup analyses by individual characteristics, we found that females and patients aged 41–65 years were more vulnerable to temperature decrease than males, patients aged 0–40 and ≧66 years, respectively.

**Conclusions:**

This study suggested that temperature decrease was a risk factor for increases in RA admission. Females and patients aged 41–65 years were particularly vulnerable to the effect of temperature decrease.

**Supplementary Information:**

The online version contains supplementary material available at 10.1186/s12889-021-11994-0.

## Background

Osteoarthritis (OA) and rheumatoid arthritis (RA) are the two most common joint disorders [1], contributed to a higher burden of disease in China and the world. According to the Global Burden of Disease (GBD) study in 2018, the worldwide prevalence of OA was 41.1‰ and the number of patients reached 303.1 million by the year 2017. Meanwhile, RA affects about 19.9 million people, which account for 2.7‰ of the total population. Between 2007 and 2017, the number of all-age years who lived with disability (YLDs) attributed to OA/RA increased by 33.5 and 31.4%, respectively [2]. In 2017, one of the three leading causes of YLD in China was musculoskeletal diseases [3]. In particular, OA and RA as the two most common musculoskeletal diseases pose major threats to healthy aging by limiting patients’ physical function, quality of life and social participation which incurred considerable economic and medical burdens to individuals, families, and governments [4]. Wu et al. [4] analyzed average annual percent change (AAPC) for OA/RA in China from 1990 to 2017, the AAPC in the age-standardized rate of disability-adjusted life years (DALYs) indicated an increasing trend for rheumatoid arthritis (0.20, 95% CI: 0.07–0.34), osteoarthritis (0.26, 95% CI: 0.20–0.31), respectively. Given the considerable disease burden of OA/RA in China, a comprehensive understanding of the risk factors for OA/RA is important for disease prevention and control. Besides genetic, immune and infectious factors, the assumption that weather influences signs and symptoms of OA and RA is widespread.

Previous studies have explored the relationship of joint pain in OA/RA with weather conditions [5–15], such as temperature and humidity, but with conflicting results [15]. For example, some studies reported that temperature decrease might influence the experience of joint pain in patients with OA/RA [5–7]. However, no significant association was observed in the findings of other studies [10, 13]. This difference may be due to a number of factors, including the diverse weather pattern in different regions, demographic characteristics and methodological limitations. In most previous studies, little attention was paid to the lagged effects of temperature change on OA/RA. Therefore, more efforts are needed to provide evidence on the risk of experiencing pain onset with temperature changes, in persons with OA/RA. In recent years, time-series analysis has been increasingly used to assess the impacts of climate change on human health. As one of the most commonly used statistical approaches in time-series analysis, a distributed lag nonlinear model (DLNM) has the merit of investigating the exposure-lag-response relationship between environmental variables and health outcomes, temperature and various diseases for example [16].

The aim of this study was to employ DLNM to examine the relationship between temperature change and outpatient admission for OA and RA in Hefei, and explore whether age or gender modified this relationship.

## Methods

### Study area

This study was conducted in Hefei, which is the capital and largest city of Anhui province in Eastern China with a population of 8.09 million inhabitants (from 2018 census data). Hefei has a humid subtropical climate with a mean temperature of 16.8 °C.

### Arthritis data

Daily counts of outpatient admission for OA/RA during 2014–2017 were obtained from The First Affiliated Hospital of University of Science and Technology of China (Anhui Provincial Hospital). The patient data included the date of outpatient admission, age, gender, residential address. Diagnosis of OA (ICD-10: M13.9) and RA (ICD-10: M06.9) was coded according to the International Classification of Disease, 10th Revision (ICD-10). Ethical approval was obtained from the Ethics Committee of Anhui Provincial Hospital prior to data collection.

### Weather and air pollutants data

Meteorological data on daily mean temperature, relative humidity, rainfall, barometric pressure and wind velocity during the same period were obtained from Hefei Bureau of Meteorology. Air pollution data including the average daily level of sulfur dioxide (SO_2_), nitrogen dioxide (NO_2_), carbon monoxide (CO), ozone (O_3_), particulate matter of less than 10 μm and 2.5 μm (PM_10_ and PM_2.5_) were collected from the Environmental Protection Bureau in Hefei. Consistent with previous study [17], we chose the 50th percentile of temperature (P50, 17.8 °C) as the reference in analyses.

### Statistical analysis

We first examined the correlations among weather indicators and pollutants with Spearman’s correlation test. Then, we applied a Poisson generalized linear regression combined with distributed lag non-linear model (DLNM) to examine the non-linear and lagged effects of ambient temperature on outpatient admission for OA/RA, after controlling for long-term trend and seasonality, day of week (*DOW*), public holidays (*Holiday*), relative humidity, wind velocity, PM_2.5_, SO_2_, NO_2_ and O_3_. The core model is expressed as follows:

$$ {Y}_t\sim Poisson\left({\mu}_t\right)\  Log\left({\mu}_t\right)=\alpha + cb\left({Temperature}_{t,l}\right)+ cb\left({Humidity}_{t,l}, df=3\right)+ cb\left({Wind}_{t,l}, df=3\right)+ cb\left({PM}_{2.5t,l}, df=3\right)+ cb\left({SO}_{2t,l}, df=3\right)+ cb\left({NO}_{2t,l}, df=3\right)+ cb\left({O}_{3t,l}, df=3\right)+ ns\left({Time}_t, df=8\right)+{\ng\mathit{DOW}}_t+{\gamma Holiday}_t+\mathrm{Lag}\left({Y}_t,1\right)+\mathrm{Lag}\left({Y}_t,2\right) $$

Where *Y*_*t*_ is the number of OA/RA admission on day *t*; *α* represents the intercept;

cb() is a cross-basis function used to models both the exposure effect and lag effect at the same time; l refers to the lag days; ns() denotes a natural cubic spline function using the dlnm package in R. To control long-term time and seasonality, we used a natural cubic spline function with 8 degrees of freedom (*dfs*) per year, along with an indicator of the day of the week (DOW) and holiday effect. A natural cubic spline with 3 *dfs* was used to for exposure dimension (mean temperature, humidity, wind velocity, PM_2.5_, SO_2_, NO_2_ and O_3_) and lag dimension (lags 0–4). In order to reduce the influence of model autocorrelation, we added autoregressive terms to the model to improve the fit of the model.

**Fig. 1:**
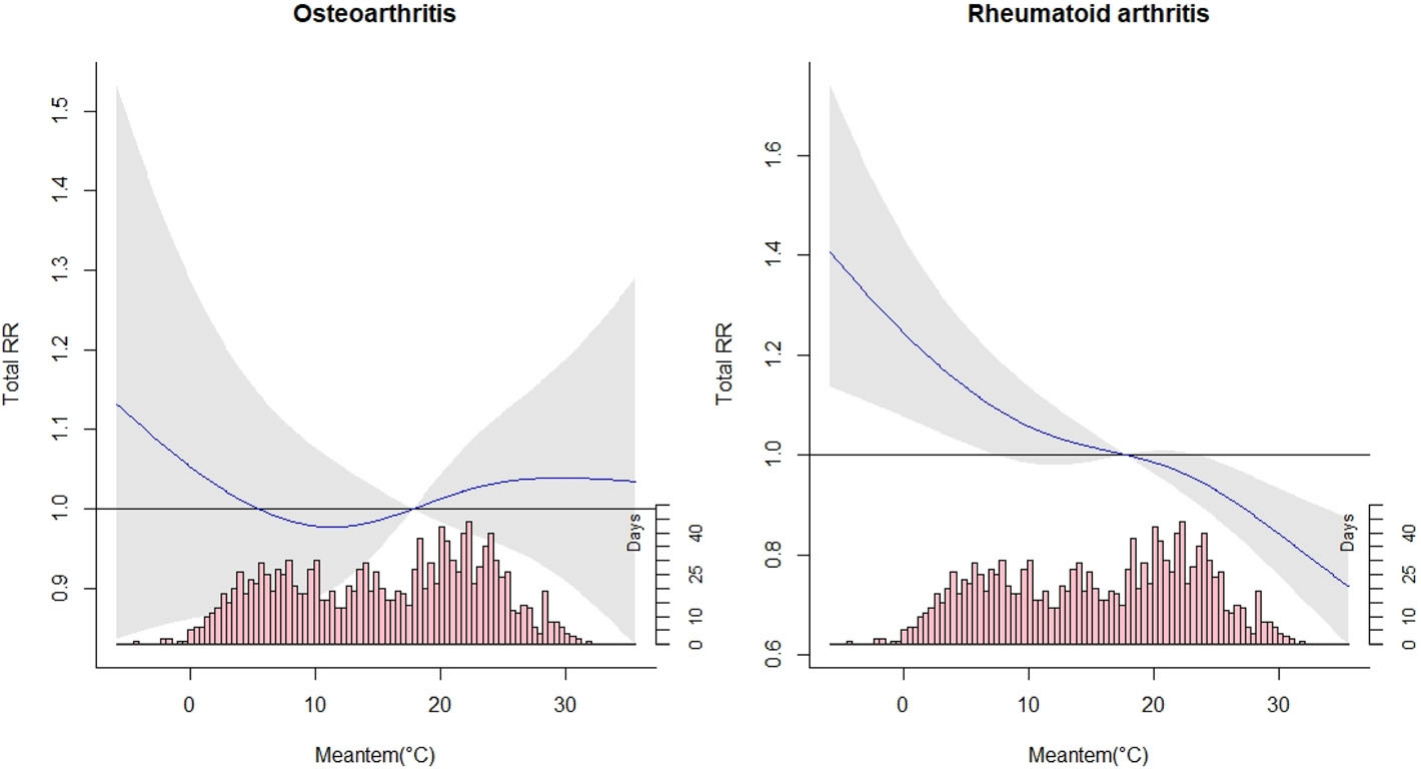
Overall effects of exposure-response associations between temperature change and outpatient admission for OA and RA

On the basis of the lowest Akaike Information Criterion (AIC), we selected the maximum lag of 4 days to capture any single and cumulative effects of temperature. Because the plot of overall exposure-response did not find the significant relationship between temperature and OA admission (Fig. 1), we only quantified the relative risks (RRs) of temperature change on RA admission by single day lags at low temperature (25th percentile, P25) compared to the reference temperature (50th percentile, P50). Furthermore, we examined the specific cumulative effects of temperature decrease on RA admission by gender (male and female) and age (0–17 years, 18–40 years, 41–65 years and ≥ 66 years). The statistically significant differences between effect estimates in subgroups were examined by the following formula:

($$ \hat{Q1} $$- $$ \hat{Q2} $$) ± 1.96 $$ \sqrt{{SE_1}^2+{SE_2}^{2.}} $$

where $\hat{Q1}$ , $\hat{Q2}$ are the estimates for two categories (such as male and female), and *SE*_1_^2^, *SE*_2_^2^ represent the corresponding standard errors [18]. The effects of temperature were estimated and reported as RR and its 95% confidence interval (CI) associated with low temperature at different lags.

To test the robustness of our results, sensitivity analyses were performed by varying *df* for time (7–9 *dfs*/year), humidity (3–5 *dfs*) and wind velocity (3–5 *dfs*), respectively. Data manipulation and analyses were conducted using R software (version 3.1.1), with the “dlnm” package to fit the DLNM [16].

## Results

### Descriptive statistics

In total, 24,838 OA cases and 43,935 RA cases were reported over the study period. For OA admission, females and patients aged 41–65 years accounted for 70.6 and 59.3%, respectively. Of RA admission, there were more female cases (36,227, 82.5%) and more cases aged 41–65 years (38,564, 87.8%). The value of daily mean temperature, relative humidity, rainfall and wind velocity were 16.8 °C, 75.5%, 3.4 mm and 1.9 m/s, respectively. The average concentration of PM_2.5_, SO_2_, NO_2_ and O_3_ during the study period were 64.6 μg/m^3^, 15.7 μg/m^3^, 39.5 μg/m^3^ and 77.9 μg/m^3^, respectively. Detailed characteristics of the study population, weather indicators and air pollutants were presented in Tables 1 and 2.

**Table 1:** Characteristics of meteorological variables and air pollutants in Hefei, China, from 2014 to 2017

Indicator	Mean ± SD	Percentile				
		5	25	50	75	95
Mean temperature (°C)	16.8 ± 9.0	2.4	8.8	17.8	24.5	30.1
Relative humidity (%)	75.5 ± 12.9	52.0	67.0	76.0	85.0	95.0
Rainfall (mm)	3.4 ± 10.1	0.0	0.0	0.0	0.9	19.8
Barometric pressure (hpa)	1015 ± 60.0	1000.1	1005.5	1013.3	1021.1	1029.1
Wind velocity (m/s)	1.9 ± 0.7	0.9	1.4	1.8	2.3	3.3
PM10 (ug/m^3^)	95.0 ± 49.1	31.0	62.0	89.0	118.0	181.9
PM2.5 (ug/m^3^)	64.6 ± 39.9	20.0	38.5	56.0	81.0	136.0
SO2 (ug/m^3^)	15.7 ± 7.2	8.0	10.0	14.0	19.0	29.0
NO2 (ug/m^3^)	39.5 ± 18.8	17.1	27.0	35.0	48.0	77.0
CO (mg/m^3^)	1.0 ± 0.3	0.6	0.8	0.9	1.1	1.6
O3 (ug/m^3^)	77.9 ± 41.9	25.0	46.0	68.0	103.0	160.0

**Table 2:** Characteristics of daily cases for OA and RA in Hefei, China, during 2014–2017

Group	Total admissions of OA/RA	Mean ± SD	Percentile				
			5	25	50	75	95
Osteoarthritis							
Total	24,838	17.0 ± 11.8	0.0	7.0	16.0	25.0	38.0
Male	7306	5.0 ± 4.0	0.0	2.0	4.0	7.0	12.0
Female	17,532	12.0 ± 8.6	0.0	5.0	11.0	18.0	27.0
< 17 years	430	0.3 ± 0.6	0.0	0.0	0.0	0.0	1.0
18 ~ years	4526	3.1 ± 2.6	0.0	1.0	3.0	5.0	8.0
41 ~ years	14,732	10.1 ± 7.4	0.0	4.0	9.0	15.0	24.0
66 ~ years	5149	3.5 ± 3.2	0.0	1.0	3.0	5.0	10.0
Rheumatoid arthritis							
Total	43,935	30.1 ± 20.1	0.0	7.0	34.0	46.0	58.0
Male	7708	5.28 ± 4.1	0.0	2.0	5.0	8.0	13.0
Female	36,227	24.8 ± 16.8	0.0	6.0	28.0	39.0	49.0
< 17 years	199	0.14 ± 0.4	0.0	0.0	0.0	0.0	1.0
18 ~ years	7297	5.0 ± 3.9	0.0	1.0	5.0	8.0	12.0
41 ~ years	38,564	19.6 ± 13.5	0.0	1.0	5.0	8.0	12.0
66 ~ years	7873	5.39 ± 4.3	0.0	1.0	5.0	9.0	13.0

### Pairwise correlation between pollutants and weather variables

Spearman’s correlation coefficients between weather variables and air pollutants in Hefei were shown in Table 3. It showed that temperature and relative humidity (*r*_*s*_ = 0.108), rainfall (*r*_*s*_ = 0.027), wind velocity (*r*_*s*_ = 0.060) were low. Meanwhile, prior studies reported that humidity increase can add to the risk of arthritis admission [8]. Thus, humidity and wind velocity were controlled for as confounders in the regression model. Similarly, PM_2.5_, SO_2_, NO_2_ and O_3_ were included in the regression model. We also observed that mean temperature was highly correlated with barometric pressure (*r*_*s*_ = − 0.906), and rainfall was highly correlated with relative humidity (rs = 0.659). Meanwhile, the correlations between PM_2.5_ and PM_10_ (*r*_*s*_ = 0.845), CO (*r*_*s*_ = 0.788) were high. Therefore, in order to avoid the multicollinearity problem, rainfall, barometric pressure, PM_10_ and CO were not included in the regression model.

**Table 3:** The Spearman correlation between weather variables and air pollutants during the study period

Indicator	Temperature	Humidity	Rainfall	Pressure	Wind velocity	PM_2.5_	PM_10_	SO_2_	CO	NO_2_	O_3_
Temperature	1.000	0.108	0.027	−0.906	0.060	−0.390	−0.183	−0.550	−0.308	−0.264	0.553
Humidity		1.000	0.659	−0.237	− 0.065	− 0.188	− 0.419	−0.405	0.028	− 0.284	− 0.292
Rainfall			1.000	− 0.172	0.175	− 0.329	− 0.487	− 0.318	− 0.175	− 0.276	− 0.225
Pressure				1.000	−0.084	0.360	0.211	0.524	0.267	0.306	−0.461
Wind velocity					1.000	−0.292	− 0.304	−0.128	− 0.399	−0.401	0.005
PM_2.5_						1.000	0.845	0.530	0.788	0.323	−0.207
PM_10_							1.000	0.585	0.682	0.435	0.012
SO_2_								1.000	0.511	0.235	−0.322
CO									1.000	0.453	−0.213
NO_2_										1.000	0.228
O_3_											1.000

### Relationship between temperature change and admission for OA and RA

Figure 1 showed the exposure-response relationship between temperature change and outpatient admission for OA and RA. It suggests that the temperature decrease was statistically associated with RA admission. In contrast, no significant association between temperature change and OA was observed. Hence, our study primarily focuses on the results of the low temperature (P25) to explore the impact of temperature decrease on the risk of RA admission.

Table 4 presents the effects of temperature decrease on RA admission over different lag days, suggesting that temperature decrease was significantly associated with increased risk of RA admission. Significant effects of temperature decrease appeared immediately and lasted about 2 days, and its effect was the greatest at the current day. Compared to the temperature of 17.8 °C, a 9 °C decrease in temperature was significantly associated with a 5.7% (1.057, 95%CI: 1.005–1.111) increase of RA admission. We also observed that females (*P* < 0.05) and patients aged 41–65 years (*P* < 0.05) were more vulnerable to the effect of temperature decrease.

**Table 4:** The effects of temperature decrease on RA admission at various lag days in Hefei, China, with 25th percentile of temperature relative to 50th percentile

Group	Lag0	Lag1	Lag2	Lag3	Lag4
Total	1.057 (1.005–1.111)*	1.044 (1.019–1.069)*	1.024 (0.986–1.064)	0.995 (0.971–1.019)	0.960 (0.913–1.010)
Male	0.994 (0.912–1.083)	1.000 (0.960–1.042)	1.007 (0.944–1.075)	1.017 (0.976–1.060)	1.028 (0.943–1.120)
Female	1.069 (1.022–1.118)*	1.030 (1.008–1.052)*	0.999 (0.966–1.034)	0.983 (0.962–1.004)	0.974 (0.931–1.019)
0–17 years	1.040 (0.662–1.633)	1.098 (0.890–1.354)	1.111 (0.795–1.552)	1.048 (0.842–1.304)	0.947 (0.609–1.473)
18–40 years	1.017 (0.924–1.119)	1.012 (0.967–1.059)	1.009 (0.938–1.085)	1.008 (0.963–1.056)	1.009 (0.917–1.112)
41–65 years	1.056 (1.005–1.110)*	1.031 (1.007–1.056)*	1.009 (0.971–1.048)	0.991 (0.967–1.015)	0.975 (0.928–1.025)
≥66 years	1.083 (0.990–1.186)	1.001 (0.960–1.048)	0.956 (0.892–1.023)	0.964 (0.923–1.006)	1.004 (0.917–1.099)

* indicates statistical significance (*P* < 0.05)

**Fig. 2:**
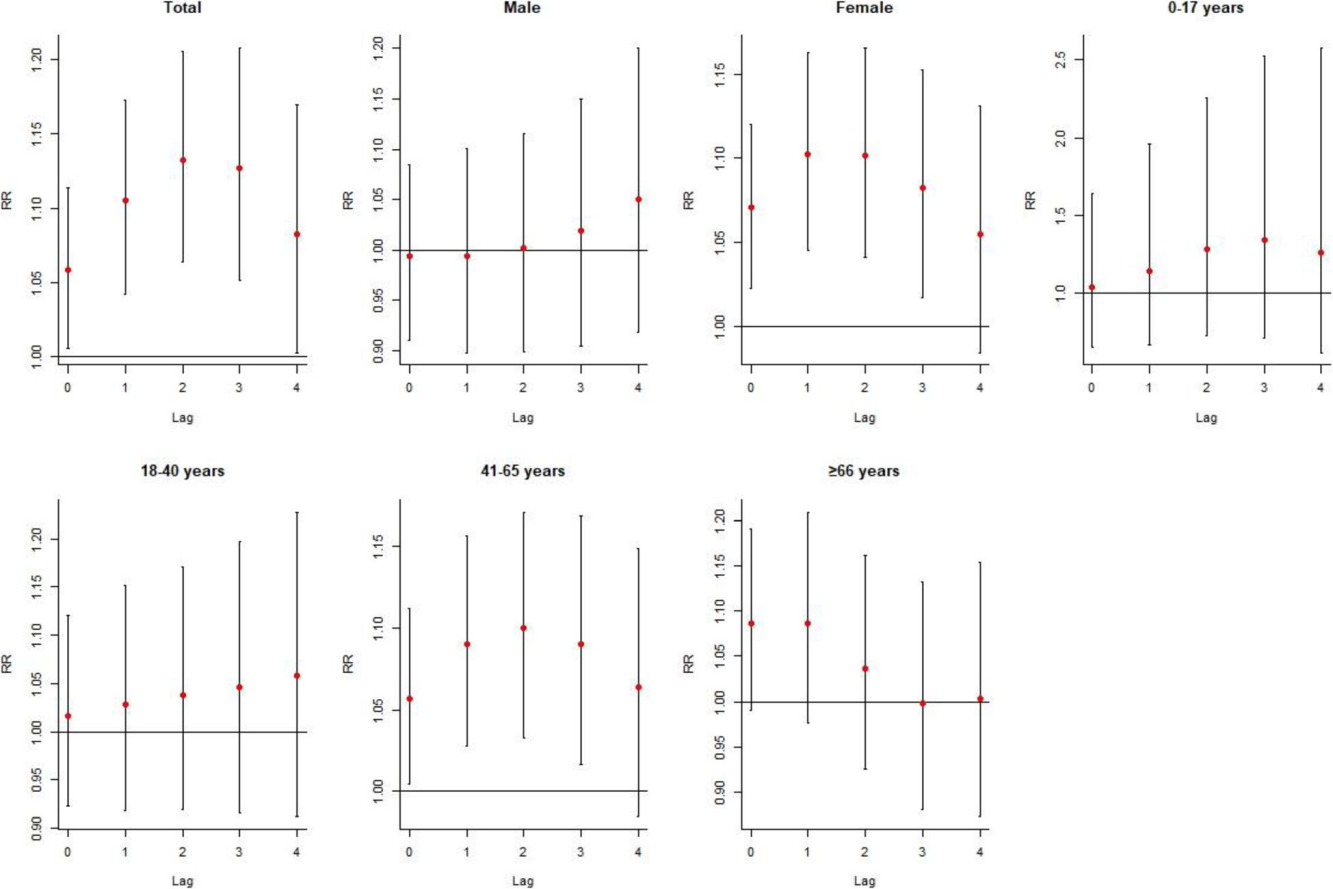
The specific cumulative effects of temperature decrease on RA admission by gender, age and classification of arthritis at different lag days, with P25 of temperature versus P50

The cumulative effects of temperature decrease on RA admission stratified by gender and age were presented in Fig. 2. It also indicated that temperature decrease was more likely to affect RA admission among females and people aged 41–65 years, with the significant multi-day RR occurring at lag0–0 and continuing to lag0–3 days. Additionally, the multi-day metrics of low temperature exposure indicated a larger and prolonged effect on RA admission than single-days exposure.

### Sensitivity analyses

To investigate whether the results were sensitive to the specification of parameters in the model, we changed *df* (7–9 per year) for time to control for the long-term trend and seasonality, and found the estimated effects of temperature did not change substantially (Supplementary Fig. S1). Moreover, we got similar results when changing *df* (3–5) for humidity and wind velocity (Supplementary Figs. S2 and S3).

## Discussion

The influence of temperature on OA/RA pain is controversial and several published studies have not demonstrated a definite relationship between temperature change and OA/RA admission. This is the first time-series study so far to quantify the associations between temperature change and OA/RA admission with a large number of patients. Our results indicate that temperature decrease has acute and delayed adverse effects on RA admission. However, the non-significant association between temperature and OA admission was observed. With regard to patients with RA, stratified analyses also provide evidence that females and patients aged 41–65 years might be more vulnerable to temperature decrease than males and patients aged 0–40, ≧66 years, respectively.

Our findings for OA might seem puzzling to some readers because it is well known that temperature can affect pain in patients with OA [6, 12, 19]. This could be caused by a cognitive misattribution. If a patient believes that cold causes arthritis pain, he or she will pay much attention to temperature decrease with much pain and go to the hospital. Meanwhile, many studies have been conducted in an attempt to establish the relationship between temperature change and OA [8, 19], but many of these studies with methodological limitations that may have to affect their validity to some extent. For instance, the study by Strusberg in the field was based on the correlation analysis [6], and this method has various shortcomings (e.g. not adjusted for confounder and not provide the value of relative risk). Although several studies have attempted to use the regression model to explore the relationship between temperature and OA [8, 20], the delayed effects of ambient temperature on OA have not been investigated. Our findings are based on the results of a time-series study and have a number of strengths compared to past research.

The traditional belief of “Cold and wet is bad, warm and dry is good for RA patients” seems to be true [21]. This study found that temperature decrease could increase risk of RA admission, which is consistent with several previous studies [5–7, 22]. The underlying mechanism as to why temperature decrease could affect RA admission is not clear enough. Abasolo et al. proposed a hypothesis that cold can trigger some diseases such as crioglobulinemia or Raynaud phenomenon, both closely related to rheumatic diseases [5]. It may be possible that muscles also play a role in relation to flares or pain in RA patients and cold weather, due to coldness stiffens muscles around the joints that can worsen the arthritis symptoms. Another explanation is the involvement of autonomic nerves to regulate the threshold of pain. An animal study by the Sato group found that both decreased temperature and air pressure led to the worsening of joint pain in arthritic rats [23]. This group also reported low temperature exposure augments pain in an animal model were mainly mediated by sympathetic nerve [24]. Nevertheless, the further studies focus on this complex area are still required.

Based on the literature review, the evidence on physiological reasons or psychological reasons for the different results between OA and RA is lacking. In Hefei, for patients who are more likely to believe that weather can influence their RA pain, the causes may be unknown, but the effect is real. Future studies are urgently needed to explore the mechanisms underlying the association between temperature decrease and increased RA admission.

Understanding the characteristics of susceptible populations is important for policy makers to develop targeted interventions [25]. A limited number of previous studies have reported that individual characteristics such as age might modify the risk of weather factors on RA disease [5]. In this study, subgroup analysis by gender found that female patients were more sensitive to temperature decrease than male patients, which might be partly to differential body composition [26]. The age-stratified analysis indicated that the association between temperature decrease and RA onset was significantly observed in patients aged 41–65 years, and this effect disappeared in other age groups. The reason might be the fact that age itself is a risk factor for disability, thus the elderly could have in general more difficulty to go to the hospital in time by themselves. Meanwhile, rheumatic pain is usually considered to be a natural part of the aging process. In order not to interfere with their study and work, young people may take other ways rather than going to the hospital to control their RA symptoms [5].

There were two major strengths in this study. To the best of our knowledge, this is the first research to quantify both lagged and non-linear relationships between temperature and RA. Additionally, our study collected 4 years data, controlled for air pollutants and explored the possible modification of confounders (e.g., gender and age) to examine the association between temperature change and RA admission. Several limitations of our study should also be noted. Firstly, the data were collected from one city, restricting our findings to be generalized to other regions of distinct weather pattern. Secondly, due to the confidentiality of information, we failed to obtain the patient data from other hospitals. Meanwhile, some patients may not go to the hospital. These factors limited us to explore the true association and may cause results bias. Thirdly, consistent with previous ecological studies, exposure misclassification should not be ignored because population exposure was used to represent personal exposure and air-conditioning was usually used in cold seasons. Fourthly, due to multiple comparisons for subgroup analyses, the type I error inflation cannot be excluded.

## Conclusions

Temperature decrease was significantly associated with an increased risk of RA admission. Females and patients aged 41–65 years were more sensitive to temperature decrease than males and other age groups, respectively. As climate change progresses, the temperature change will be more frequent, and patients may be at greater risk of RA onset associated with temperature decrease. Our findings highlighted that the public health sector, medical staff and carers of RA patients should pay attention to temperature decrease when controlling and preventing RA onset.

## Supplementary Information

**Additional file 1: Fig. S1.** The overall effects of temperature change on OA/RA admission when changing the *df* (7–9 *df*/year) for time.. **Fig. S2.** The overall effects of temperature change on OA/RA admission when varying the *df* (3–5) for relative humidity. **Fig. S3.** The overall effects of temperature change on OA/RA admission when varying the df (3–5) for wind velocity.

## Data Availability

The data analyzed during the study are not publicly available due to the data also forms part of an ongoing study but are available from the corresponding author on reasonable request.

## Abbreviations

AAPC: Average annual percent change
AIC: Akaike Information Criterion
CO: Carbon monoxide
*dfs*: Degrees of freedom
DALYs: Disability-adjusted life years
DLNM: Distributed lag nonlinear model
DOW: Day of week
GBD: Global Burden of Disease
ICD: International Classification of Disease
NO_2_: Nitrogen dioxide
OA: Osteoarthritis
O_3_: Ozone
PM_10_: Particulate matter of less than 10 μm
PM_2.5_: Particulate matter of less than 2.5 μm
RA: Rheumatoid arthritis
RR: Relative risk
SO_2_: Sulfur dioxide
YLD: Years lived with disability.
