# Supplementary figures and images for: Effect of ambient temperature on outpatient admission for osteoarthritis and rheumatoid arthritis in a subtropical Chinese city
Source: BMC Public Health. 2022 Jan 25;22:172. doi: 10.1186/s12889-021-11994-0 (PMC8790907; doi:10.1186/s12889-021-11994-0)

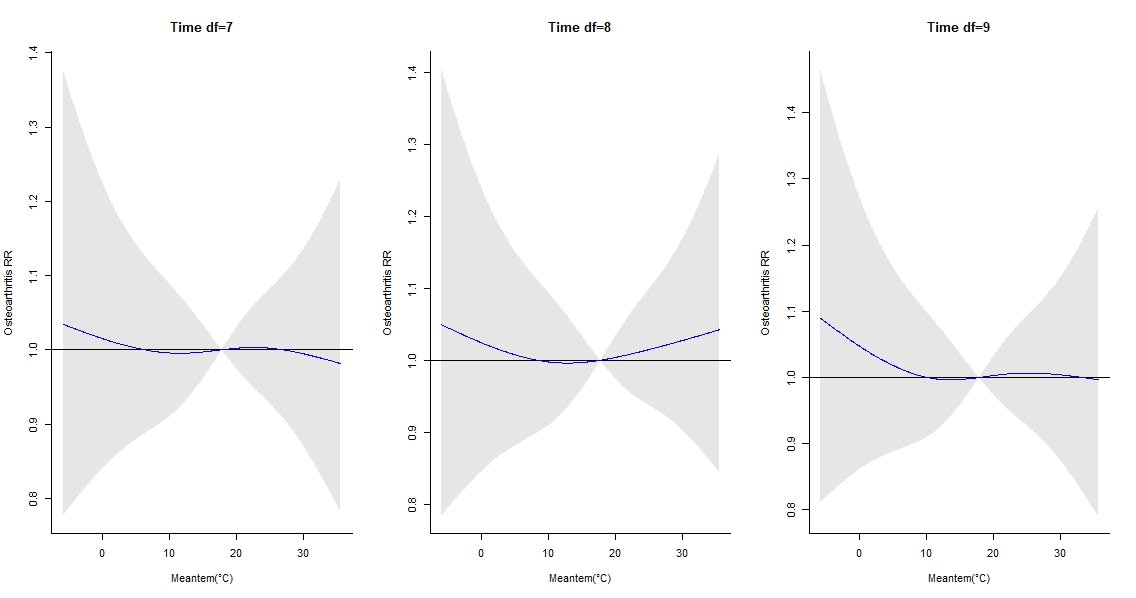


**Figure S1**


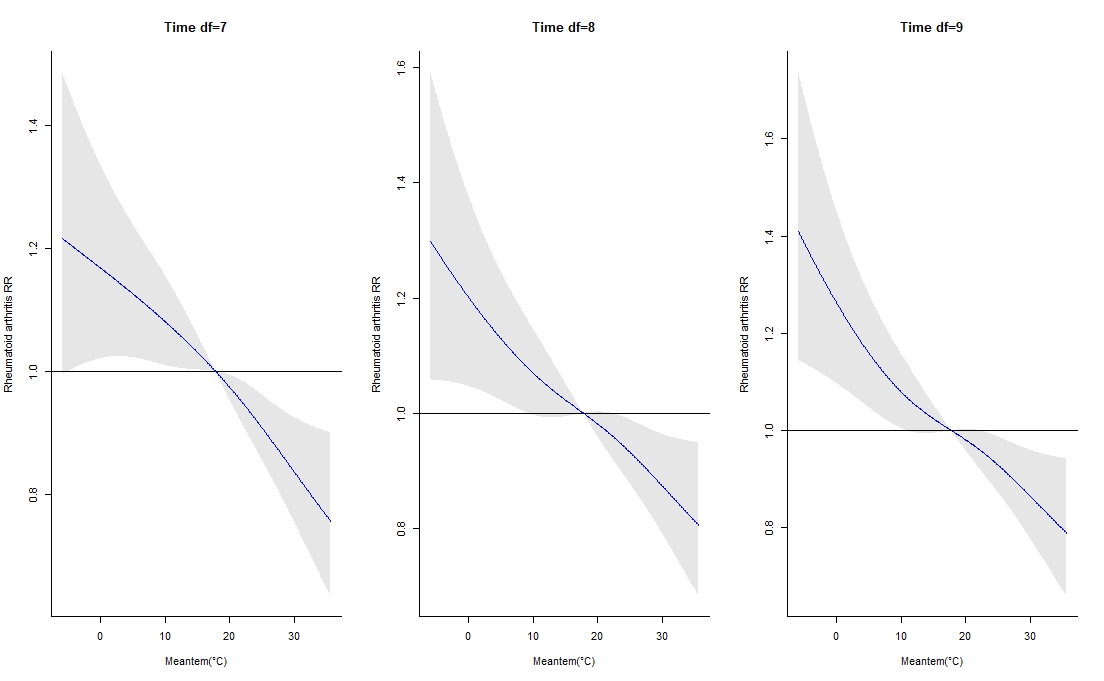

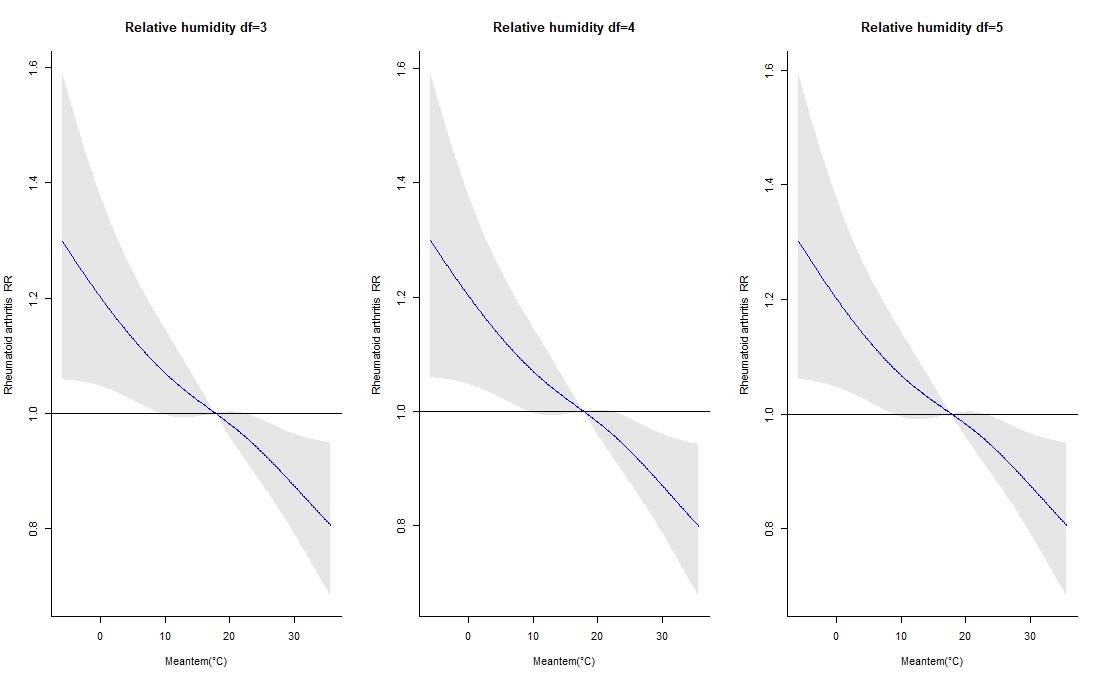

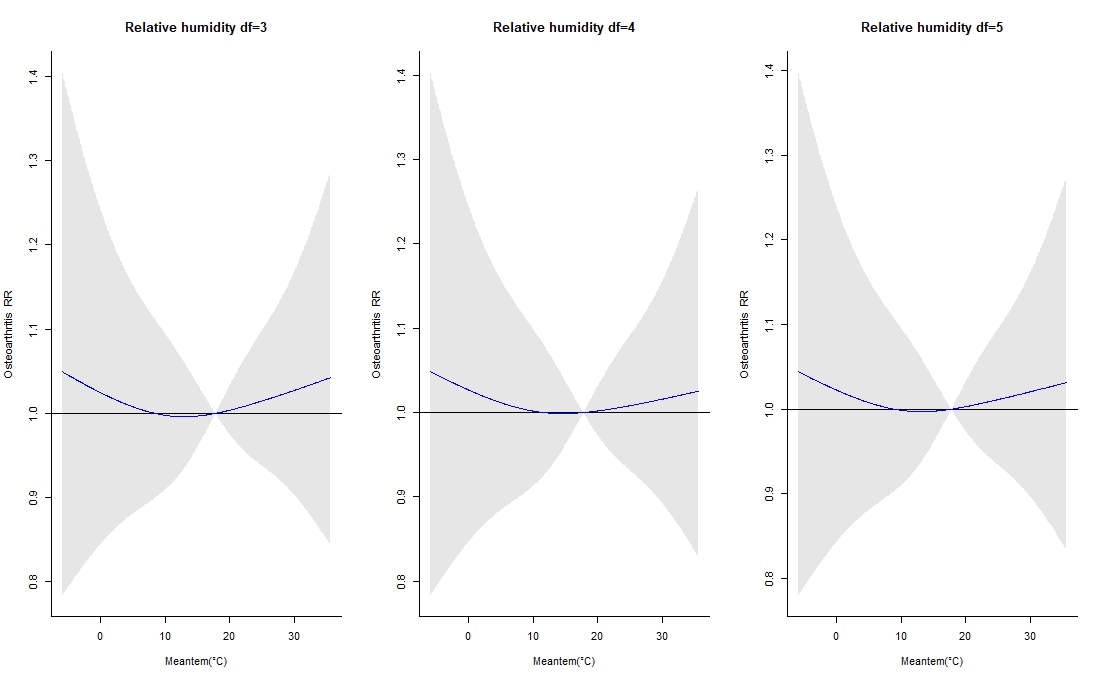


**Figure S2**


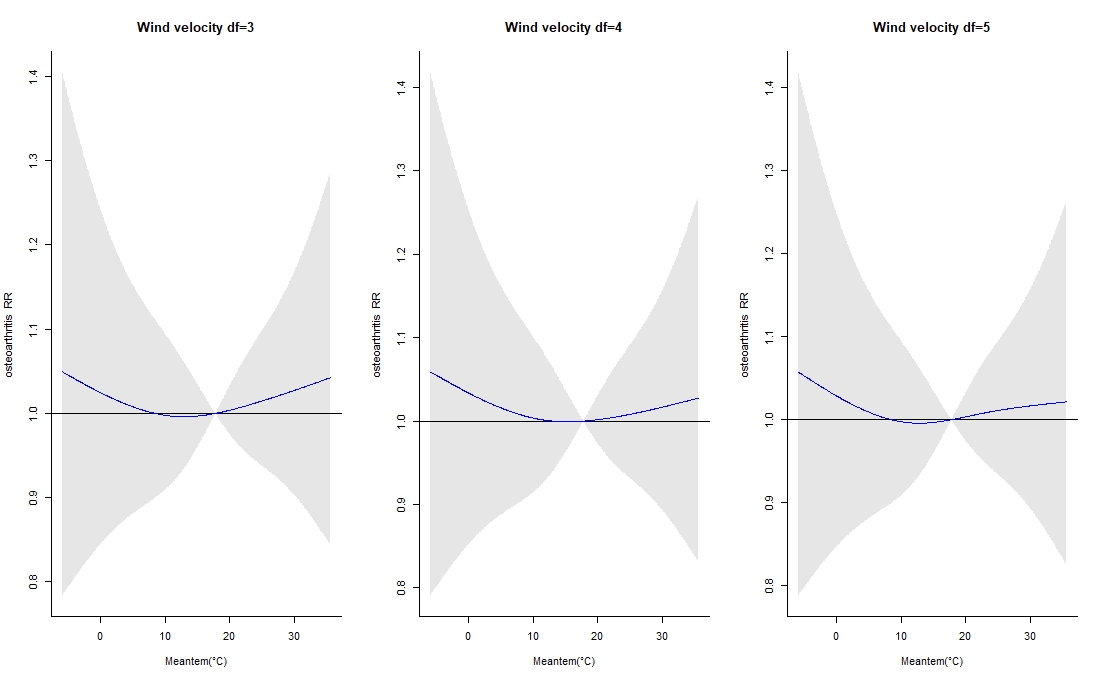

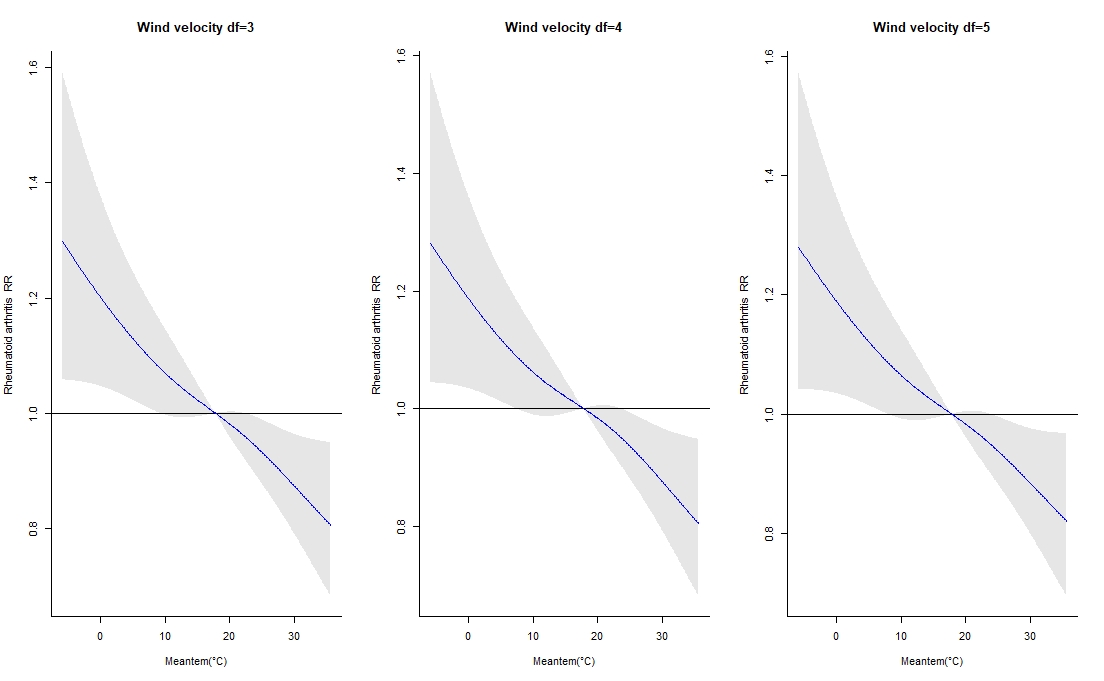


**Figure S3**

Supplement: Supplementary file 1 — Additional file 1: Fig. S1. The overall effects of temperature change on OA/RA admission when changing the df (7–9 df/year) for time.. Fig. S2. The overall effects of temperature change on OA/RA admission when varying the df (3–5) for relative humidity. Fig. S3. The overall effects of temperature change on OA/RA admission when varying the df (3–5) for wind velocity. [file 12889_2021_11994_MOESM1_ESM.doc]
